# Adaptive Beamforming for On-Orbit Satellite-Based ADS-B Based on FCNN

**DOI:** 10.3390/s24217065

**Published:** 2024-11-02

**Authors:** Yiran Xiang, Songting Li, Lihu Chen

**Affiliations:** College of Aerospace Science and Engineering, National University of Defense Technology, Changsha 410073, China; x_yiran22@nudt.edu.cn (Y.X.); clh2055@163.com (L.C.)

**Keywords:** satellite-based ADS-B, fully connected neural network, adaptive beamforming

## Abstract

Digital multi-beam synthesis technology is generally used in the on-orbit satellite-based Automatic Dependent Surveillance–Broadcast (ADS-B) system. However, the probability of successfully detecting aircraft with uneven surface distribution is low. An adaptive digital beamforming method is proposed to improve the efficiency of aircraft detection probability. The current method has the problem of long operation time and is not suitable for on-orbit operation. Therefore, this paper proposes an adaptive beamforming method for the ADS-B system based on a fully connected neural network (FCNN). The simulation results show that the calculation time of this method is about 2.6 s when more than 15,000 sets of data are inputted, which is 15–80% better than the existing methods. Its detection success probability is 10% higher than those of existing methods, and it has better robustness against large amounts of data.

## 1. Introduction

Automatic Dependent Surveillance–Broadcast (ADS-B), a next-generation surveillance technology endorsed by the International Civil Aviation Organization (ICAO), seamlessly integrates surveillance and communication capabilities. Aircraft equipped with this system automatically transmits flight numbers, positions, and other information. Aircraft and base stations with receivers process the transmitted messages to support the global Air Traffic Management (ATM) system [1,2]. Due to geographical and political factors, deploying terrestrial-based ADS-B base stations has limitations in achieving global coverage. In response to the demand for real-time information return and seamless ground coverage within the ATM system, the ICAO introduced a satellite-based ADS-B system. This system offers extensive real-time coverage with a wide geographical reach and minimal layout restrictions. The PROBA-V satellite [3] of the European Space Agency (ESA), the GOMX series satellite [4] of Denmark, the TianTou series satellite [5,6] of China, the KS-1 satellite [7], and the UST-2 satellite [8,9] of China have verified the feasibility of carrying ADS-B on a single satellite. The Iridium II satellite program [10], the Skywalker constellation of China and the German Aerospace Center [11], the China Satellite Network Group Co., and other companies rely on the constellation for system construction.

**Figure 1:**
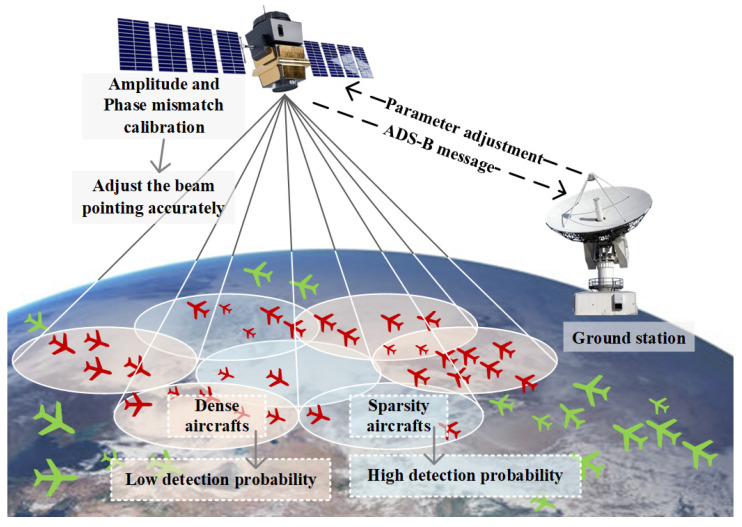
Principle of adaptive beamforming for on-orbit satellite-based ADS-B.

However, it also presents several challenges, including longer star-ground propagation paths, significant path loss, unequal coverage between star-carried receivers and ground base stations, and prominent multi-signal conflict issues within the system [12,13]. To address these challenges, Iridium II and the China Satellite Network Group Co., Ltd., developed a receiver with a digital phased array multi-beam antenna to enhance gain while effectively upholding overall coverage. In digital beamforming, the amplitude and phase mismatch will occur among the Radio Frequency (RF) receiving channels, resulting in lower antenna gain and beam deflection [14]. When calibrating the amplitude–phase mismatch on satellites, the analytical signals of all channels and reference channels are compensated by the compensation parameters in the amplitude and phase calibration module of the receivers [15,16,17]. This method is simple but requires recalibration when repowering. In this paper, a new amplitude–phase mismatch calibration technique based on the Least Mean Square (LMS) algorithm is proposed, which solves the shortcomings of existing methods and can be adapted to real-time calibration. Amplitude and phase mismatch calibration technology is an essential prerequisite for successful beamforming. Only after amplitude–phase calibration can the beam be pointed accurately, laying a good foundation for the next step of adaptive beamforming. Figure 1. shows the principle of adaptive beamforming for on-orbit satellite-based ADS-B.

The adaptive beamforming method is designed to adjust the beam pattern dynamically according to the aircraft distribution on the surface to reduce signal collisions in the sub-beams. In [18], an adaptive beamforming method based on the genetic algorithm is proposed, which improves the aircraft detection probability by adjusting the direction of the sub-beams. This method uses the genetic algorithm to iteratively optimize the amplitude and phase of the antenna array element to adjust the direction of the sub-beams. In [19], a beamforming method using distributed cooperative coevolution with an adaptive grouping strategy is introduced, effectively enhancing the aircraft’s convergence velocity and detection probability. Although the existing methods effectively improve the probability of aircraft detection, they have the problems of long operation time and excessive occupation of resources on the satellite. Due to the short transit time of satellites’ communication links and the limited computing resources of onboard computers, the existing methods cannot be implemented in engineering. Faced with such a dilemma, this paper proposes an on-orbit adaptative beamforming method based on FCNN. Based on the detection of ADS-B signals furnished by TianTuo series satellites, an adaptive beamforming dataset is established, and an adaptive beamforming method based on a fully connected neural network (FCNN) is proposed. The six-layer FCNN is trained with the constructed dataset, and the accuracy of the test result exceeds 97.2%.

The structure of this paper is arranged as follows: In Section 1, the introduction section first introduces the difficulties faced in the development process of the spaceborne ADS-B system. Aiming at the problem of low aircraft detection probability caused by the uneven distribution of surface aircraft, this section mainly introduces the research status of the beam adaptive adjustment method at home and abroad. In Section 2, the system is modeled by the surface aircraft distribution. The optimization objective of the subsequent optimization dissemination is determined as the aircraft detection probability. In addition, this chapter also constructs the on-orbit beam number adaptive implementation architecture. Section 3 introduces the amplitude–phase mismatch calibration technology based on the LMS algorithm, and the beam pointing after amplitude–phase mismatch calibration is more accurate. Then, according to the satellite downlink data, the beam adaptive dataset is established to prepare for the next step of training the neural network. Finally, the adaptive beam adjustment method based on FCNN is described. In Section 4, the proposed method is simulated. The last chapter summarizes the whole paper and prospects for future work.

## 2. Aircraft Detection Probability Model

The satellite-based ADS-B system requires the reception of a complete and processable message to monitor ground targets effectively. This message must be transmitted error-free in free space, without any symbol errors, while ensuring no signal conflicts occur within the visual range of the satellite when multiple target messages are present [20]. As the system uses the Aloha protocol, the ADS-B signal transmission model is established based on the Poisson distribution. Take the expected value of data transmitted per unit of time as the load of the Poisson process, which can be written as follows:

(1) $$G=N\cdot \tau \cdot v_{tx}$$

where N denotes the number of aircraft in the coverage area, τ denotes a unit of time, and $v_{tx}$ denotes the frame rate of the transmitter. Then the probability of producing k packets in the t frame time is as follows:

(2) $$P\left(k,t\right)=\frac{{\left(tG\right)}^{k}}{k!}\cdot e^{-tG}$$

Currently, ground-based equipment can separate overlapping packets [21]. Still, due to the limited computing power resources on the satellite, the ADS-B receiver on the satellite cannot separate aliasing signals. The space-based ADS-B receiver cannot separate aliased signals, so two packets will be dropped simultaneously when packet overlap occurs in the receiving channel. Therefore, in the modeling process, it is considered that the signal transmission is successful if and only if no other signals are detected within two frames. Substituting t=2 and k=0, the signal unpacking rate $P_{n}$ is obtained as follows:

(3) $$P_{n}=\frac{{\left(2\cdot G\right)}^{0}}{0!}\cdot e^{-2G}=e^{-2G}$$

The signal operating frequency band at 1090 MHz includes the 1090ES protocol adopted by ADS-B and the Mode-S short reply signal. The frame lengths of ADS-B signal and Mode-S signal are $\tau_{\mathrm{ADS}\text{-}B}=120\;\mu s$ and $\tau_{\mathrm{Mode}\text{-}S}=64\;\mu s$ [22]. Modify the model, substitute $K_{\mathrm{ADS}\text{-}B}=2$ and $K_{\mathrm{Mode}\text{-}S}=1.5$, and then the corrected signal unpacking rate $P_{\mathrm{ne}}$ is as follows:

(4) $$P_{\mathrm{ne}}=e^{-(K_{\mathrm{ADS}\text{-}B}+K_{\mathrm{Mode}\text{-}S})G}=e^{-3.5G}$$

ADS-B signal contains a 24-bit CRC check code, so the packet can be successfully transmitted when the number of bit errors is fewer than or equal to 5. Taking the bit error rate $P_{b}=10^{-3}$, the probability of a successful packet transmission is as follows:

(5) $$P_{s}=\sum_{n=0}^{5}C_{112}^{n}{P_{b}}^{n}{\left(1-P_{b}\right)}^{112-n}$$

When the packet is successfully transmitted and correctly unpacketized, the detection probability of each packet is as follows:

(6) $$P_{d}=P_{s}\cdot P_{\mathrm{ne}}$$

According to the requirements of the air traffic control system, the concept of system detection probability $P_{\mathrm{UI}}$ is introduced. The sending rate of the ADS-B position message f=1 Hz; in $T_{\mathrm{UI}}$ time, the system obtains the position of the aircraft with a probability of not less than $P_{\mathrm{UI}}$, which needs to be satisfied, as follows:

(7) $$\left(1-P_{\mathrm{UI}}\right)={\left(1-P_{d}\right)}^{T_{\mathrm{UI}}\cdot f}$$

The air traffic control industry currently requires a receiver’s correct unpacking rate $P_{\mathrm{UI}}$ of at least 95%. For the terrestrial-based ADS-B system, the data refresh time interval $T_{\mathrm{UI}}$ should not exceed 4 s, while for the satellite-based ADS-B system, it should not exceed 8 s. Additionally, the maximum number of visible aircraft within a single beam can be calculated as no more than 617.

Based on the ADS-B message, data are received by the TianTuo series satellites of the National University of Defense Technology (NUDT) on orbit over recent years. In [23], a visualization processing model for aircraft distribution based on the message is proposed. The satellite-based ADS-B receiver receives, processes, decodes, and forwards the received ADS-B messages. The ground station receives 28-bit hexadecimal messages, including information such as time stamp, DF code, AC code, flight number, flight latitude, and longitude. This study only focuses on the distribution of aircraft, so redundant data are removed to reduce the dimension of message information. The two-dimensional information of the longitude and latitude of flights is taken as a sample of the visual processing model, and Earth’s surface is divided into cells with 1° longitude and latitude as a step. The number of aircraft that can communicate there is screened by considering the antenna elevation information of each cell on the surface. To improve the universality of data, the messages received by the TianTuo series satellites in the past three months are accumulated. The projection position of the aircraft is marked and processed on the map according to the message information, and the airflow density diagram is obtained. The maximum number of aircraft per unit step cell is 4500. Civil flights have the characteristics of relatively fixed routes and stable aircraft distribution over an extended period. Therefore, this design assumes that the aircraft message information accumulated in three months can better describe the surface route and aircraft distribution.

**Figure 2:**
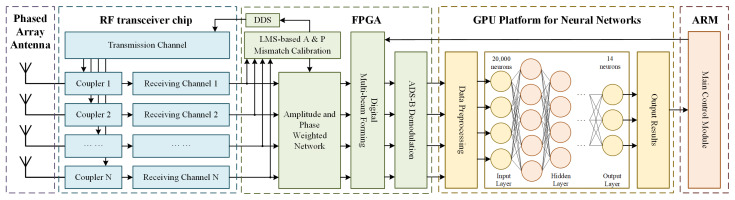
Flow diagram of the on-orbit ADS-B adaptive beamforming method.

In this paper, we illustrate the budgeting of communication links using 11 Walker constellations at a height of 780 km per 6 orbits. Due to the extensive propagation distance between the satellite and Earth, the receiving power of a single beam cannot exceed the receiver sensitivity by more than 3 dB to establish stable communication links. Given the limited space on satellite platforms, a digital multi-beam phased array antenna is employed to achieve this. Using split beams effectively reduces beam width and increases overall gain while maintaining unchanged coverage. Therefore, the whole flow diagram of the on-orbit ADS-B adaptive beamforming method is established, as shown in Figure 2.

The phased array antenna captures the ADS-B packet from surface aircraft, and the RF front end’s received signal undergoes filtering, amplification, and down-conversion to yield the baseband signal. First, the multi-path baseband signal input Field Programmable Gate Array (FPGA) is calibrated for amplitude–phase mismatch to ensure accurate beam pointing and correct ADS-B packet demodulation. After multi-channel amplitude–phase mismatch calibration, one transmitting channel inputs the calibration information into the couplers to compensate for each receiving channel. After calibration, multi-beamforming and ADS-B packet demodulation are performed before the packet is fed into the GPU platform for the neural network. The demodulated packets are processed, and the longitude and latitude information of the aircraft is obtained. Input the longitude and latitude information into the trained neural network to calculate an adaptive beamforming scheme. According to the scheme, the main control module, ARM, adjusts the sub-beam direction to improve the detection probability of aircraft.

## 3. Adaptive Multi-Beamforming

### 3.1. Adaptive Amplitude–Phase Mismatch Calibration

The satellite-based ADS-B system employs digital beamforming (DBF) technology, where each antenna element is connected to a separate RF channel. Due to random errors in PVT (process, voltage, temperature), amplitude and phase mismatch errors occur between channels, resulting in a shift in the synthesized beam direction and a decrease in gain. Therefore, a phase and amplitude mismatch calibration technique is proposed based on the LMS algorithm. The gradient of the square of a single error sample is used to estimate the mean square error gradient without requiring knowledge of the statistical characteristics of the input and desired signals. The weight coefficient of the current moment is obtained by adding the proportional term of the negative mean square error gradient to the weight coefficient of the previous moment.

During the phase mismatch calibration, a single-tone signal is sent to all receiving channels simultaneously to ensure that the transmission distance from the transmitting signal to each receiving channel is equal. Then, one of the receiving channels is selected as the reference channel, and the remaining channels are calibrated sequentially with the reference channel using the LMS algorithm. The LMS algorithm is a widely used adaptive filtering algorithm. The basic iterative process of the LMS algorithm is to loop the following three steps in which $y\left(n\right)$ is infinitely approximated by $d\left(n\right)$, with $e\left(n\right)$ gradually decreasing, $e\left(n\right)*$ is the transpose of $e\left(n\right)$

- Filter: $y\left(n\right)=\omega *\left(n\right)\cdot x\left(n\right)$
- Error calculations: $e\left(n\right)=r\left(n\right)-y\left(n\right)$; $\nabla \left(n\right)=x\left(n\right)e\left(n\right)*$
- Update the filter parameters: $\omega \left(n+1\right)=\omega \left(n\right)+2\mu \nabla \left(n\right)$

**Figure 3:**
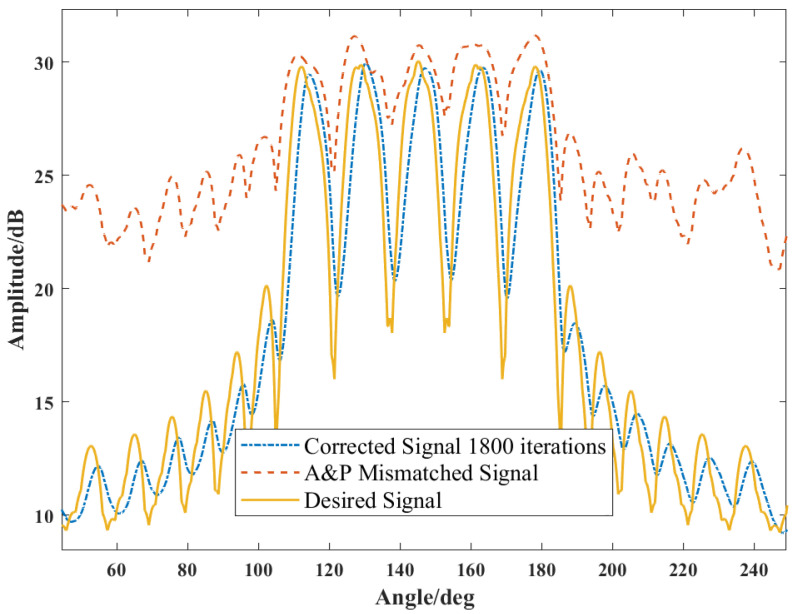
LMS-based amplitude–phase mismatch calibration.

The adaptive amplitude–phase mismatch calibration can effectively improve the beam gain, and accurate beam pointing is a necessary precondition for the adaptive adjustment of the subsequent beams. Taking the amplitude–phase mismatch calibration of a 25-channel synthetic 5-beam as an example, random noise is added to each channel to obtain the amplitude–phase mismatch signal. An example of calibrated and uncalibrated beams is shown in Figure 3.

### 3.2. Adaptive Beamforming Model Design

Due to the uneven distribution of aircraft on the surface, the partial beam coverage of aircraft exceeds 361, which is insufficient to meet the engineering and commercial demand for spaceborne ADS-B. Therefore, it is proposed to adaptively adjust the pointing width of the sub-beam according to the aircraft distribution: reducing the sub-beam radius in dense areas of aircraft to decrease the number of covered targets while improving beam gain, increasing the sub-beam radius in sparse areas of aircraft, and adjusting sub-beam pointing to ensure overall beam coverage.

**Figure 4:**
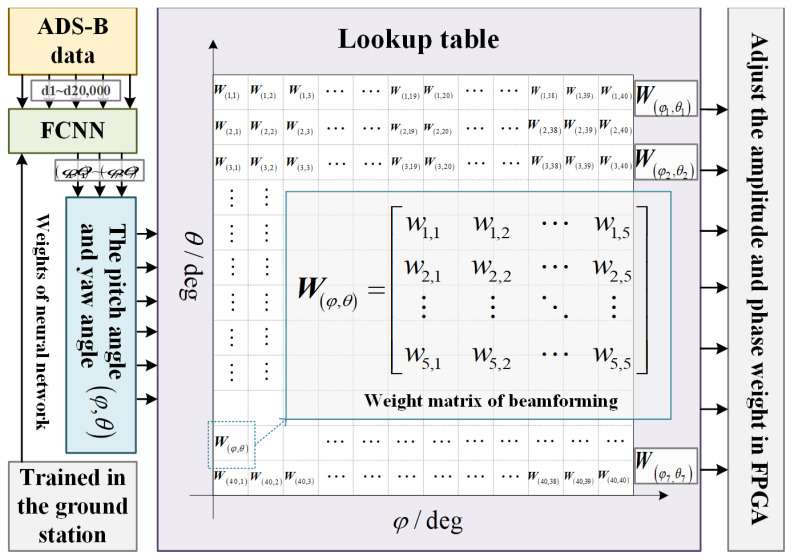
The implementation process of an adaptive beamforming scheme.

The current adaptive beamforming algorithms aim to minimize the missed detection probability of aircraft. They utilize genetic algorithms, co-evolutionary algorithms, and other methods to optimize the objective function and then adjust the beam direction and radius. However, these methods are hindered by a large amount of calculation and the inability to respond quickly, which is not conducive to satellite implementation. This paper proposes using machine learning to construct a neural network that rapidly responds to ground targets. The network will be trained with input data in advance to achieve a fast response to the distribution of input ground targets and an adaptive output beamforming scheme. This design inputs the aircraft’s position information into the neural network and outputs the pitch angle and yaw angle of the sub-beams. The range of the pitch angle and yaw angle, which is between +180 deg and −180 deg, is divided into 40 subintervals. Each subinterval corresponds to a weight matrix of beam synthesis, and such a one-to-one correspondence lookup table is established in the ARM, as shown in Figure 4. Input the adjusted the pitch angle and yaw angle to the ARM, and the ARM outputs the beam synthesis weight matrix against the lookup table. The matrix is fed into the beam synthesis module of the FPGA, and the multiplier is used to adjust the beam direction to achieve the adaptive adjustment of beam direction on the satellite.

### 3.3. Construction of Beam Adaptive Dataset

The TianTuo series satellites can detect ADS-B messages transmitted by ground aircraft during operation and obtain the ground aircraft’s basic distribution after screening and decoding. In this study, the actual distribution of ground aircraft from March to August 2022 is obtained based on satellite downlink data. Furthermore, a dataset for the adaptive beamforming method is constructed based on the real data.

Using the data obtained from the reconnaissance mission in May 2022 as an example, the satellite received 1,048,576 usable packets. Upon decoding, the multi-dimensional flight information was reduced to only retaining two-dimensional information regarding flight latitude and longitude. It can be inferred that the beam covers a range of approximately 40° in latitude and longitude. Therefore, the longitude and latitude information of the flight is initially divided into intervals of 40°, allowing for the determination of aircraft distribution within the visible range of the beam. Ten thousand two-dimensional datasets were randomly selected as input from the initial sample set. Subsequently, a genetic algorithm adaptively adjusted seven sub-beams with a fixed uniform distribution. The pitch angle and yaw angle of these seven sub-beams, after adaptive adjustment, could then be outputted because the longitude and latitude information of the input aircraft is an absolute value, while the pointing angle of the output beam is a relative value. It is necessary to normalize the input data from absolute value to relative value to enhance the training accuracy of the neural network. This normalized input and output dataset is then used as a sample for training the neural network. In this paper, a total of 300 original datasets are established through the decoding, dimension reduction, partitioning, sampling, and reconstruction of messages received by TianTuo series satellites in May 2022. The datasets include 255 groups for training and 45 groups for testing.

### 3.4. Design of Beam Adaptation Algorithm Based on FCNN

Due to the high velocity of the satellite, ground scanning is conducted rapidly. However, the limited computing resources on the satellite are insufficient to support adaptive beamforming calculations based on genetic and co-evolutionary algorithms. Therefore, this study proposes an adaptive beamforming method based on a neural network.

The artificial neural network is an abstract mathematical model that mimics biological neurons and processes data by simulating the learning process of the biological brain. It is suitable for solving nonlinear and fuzzy model feature problems [24,25,26]. In this study, a fully connected neural network is utilized, where the nodes of each layer are interconnected with every node of the adjacent layer. The aircraft position information matrix is transformed into 1 × 20,000 discrete data, and the normalized aircraft position serves as the input layer. Simultaneously, the matrix containing the pitch and yaw angles of seven sub-beams in a 1 × 14 format is integrated as the output layer to construct the fully connected neural network. Each neuron conducts linear processing of the input value through weight and bias, which can be simulated by a linear equation, $Z=\omega X^{T}+b$, where ω represents weight and b represents bias. The loss function used in the process of network backpropagation is the MSE function, and the expression is as follows:

(8) $$MSELoos=\frac{1}{N}\sum_{i=1}^{N}{\left(y\_true_{i}-y\_pred_{i}\right)}^{2}$$

(9) $$MSE\_mean=\frac{1}{M}losses$$

where $y\_true_{i}$ represents the real data, $y\_pred_{i}$ represents the predicted data, and losses represent the cumulative value of the loss function. Add the activation functions ReLU and tanh to the hidden layer to reduce computational complexity and alleviate gradient vanishing and overfitting. The expressions are as follows:

(10) $$\mathrm{ReLU}\left(x\right)=\max\left(0,x\right)$$

(11) $$\tanh\left(x\right)=\frac{e^{x}-e^{-x}}{e^{x}+e^{-x}}$$

## 4. Simulation Results

To test the ability of the trained neural network to adjust the beam, the training results of the neural network are vertically tested in this part from model calibration accuracy and model convergence speed. Then, the calculation time and optimization ability of the neural network-based beam adaptive adjustment method are compared with the existing methods.

**Figure 5:**
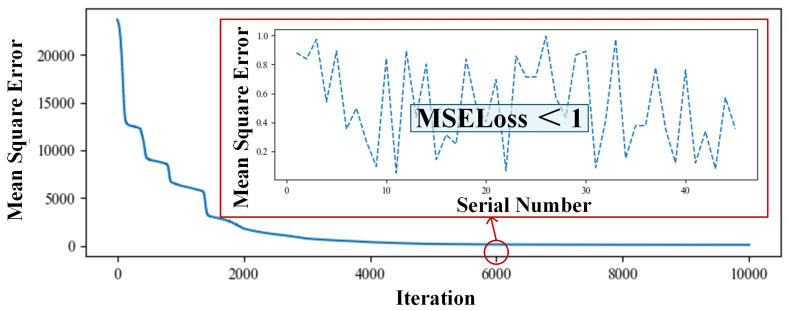
The result using the weighted network of the 6000th iteration.

This design simulates the phased array antenna with seven sub-beams. The neural network is trained by the dataset constructed in Section 3. When the number of network layers is fewer than four network layers, the convergence algebra is too high, and the result often appears to be the problem of underfitting, which is not suitable for adaptive beamforming requiring fast response. When the number of layers is higher than eight, the convergence algebra decreases significantly, but more than 50% of the training results will show an overfitting phenomenon. Therefore, this design builds the six-layer fully connected neural network, and the number of neurons in the middle five hidden layers is set to 16,384, 8192, 2048, 1024, and 256. The change curve of the loss function, as shown in Figure 5, can be obtained when the number of cycle times is 7000. In order to more clearly observe the average variation trend of the loss function, this study accumulates the loss functions of all training sets to obtain overall losses. Therefore, based on the calculations in Equations (2) and (3), it can be inferred that as the training results tend towards stability, the mean square error for each group of training results is approximately 0.2998. This model utilizes a six-layer neural network, and the training results typically converge after 5000 epochs on average. The performance of this neural network is evaluated using the test set, and the situation where the test set is tested using the weighted network of the 6000th iteration is shown in Figure 5 and the loss of the test set is less than 1.

**Figure 6:**
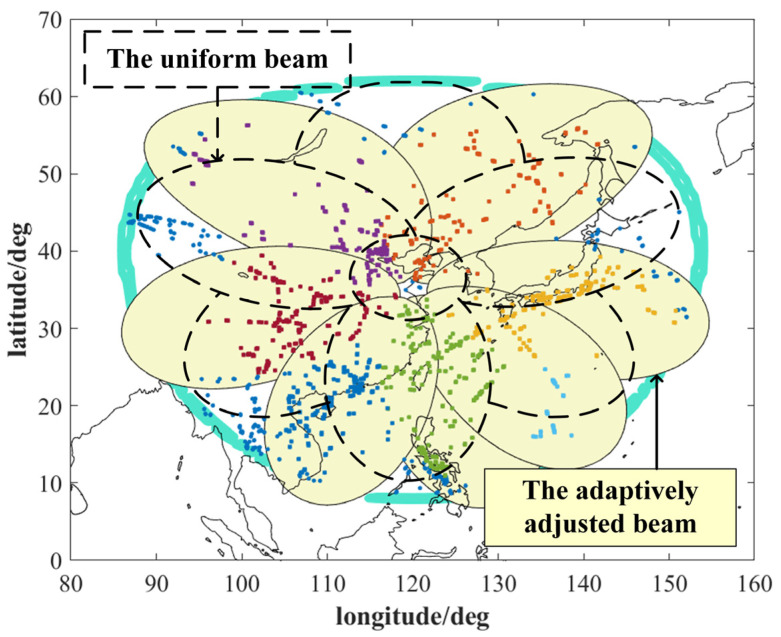
The uniform beam and the adaptively adjusted beam.

**Figure 7:**
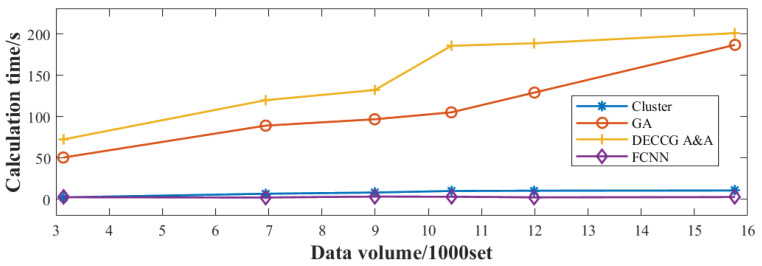
Calculation time comparison chart.

**Figure 8:**
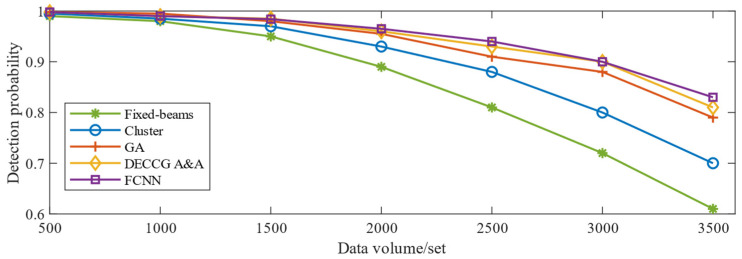
Detection probability of aircraft comparison chart.

A comparison between the adaptively adjusted beam and the uniform beam is shown in Figure 6, and the detection probability of aircraft is increased by 15.68%. To further validate the adjustment effect of the neural network-based adaptive beamforming method, the same test data are inputted into the horizontal comparison calculation time and optimization ability of the four adaptive beamforming methods based on a neural network, clustering algorithm, genetic algorithm, and coevolutionary algorithm. The results are obtained in Figure 7. Figure 7 shows that the computation time of the beam adaptive adjustment technique based on FCNN is reduced by 15~80% compared with that based on Cluster, GA, and DECCG&AA. Moreover, the method has good robustness, and the calculation time does not increase significantly with the increase in the number of data groups. While the pitch angle and yaw angle of the beam are output, the number of aircraft in each sub-beam can be determined according to the new pointing of the beam to calculate the aircraft detection probability by using Formula (7). The comparison diagram intuitively demonstrates that the neural network-based beam adaptive method enhances aircraft detection probability by 10% to 80% in computation time compared with other methods. As shown in Figure 8, the optimization results show a similar improvement to the genetic algorithm-based beam adaptive method. They are significantly higher than those of the cluster-based beam adaptive method. Figure 8 shows that the probability of aircraft detection is greatly affected by the increase in the number of data groups. Furthermore, the neural network-based beam adaptive method exhibits good robustness in handling increased data volume, indicating stronger computing power than the other three algorithms.

## 5. Conclusions

In this paper, an adaptive beamforming algorithm based on a fully connected neural network is designed for multiple sub-beams of the ADS-B system. Using the real message data collected by TianTuo series satellites on orbit, a sample set is constructed to train and test the six-layer fully connected neural network. The ground station can use the trained neural network to quickly process the ADS-B message transmitted by the satellite in real time, obtain a feasible adaptive beamforming scheme, and inject the satellite. It solves the problem of insufficient computing resources on board, slow beam adaptive response, and inability to adjust the beam to the distribution of ground aircraft. The following question may be considered in the next step of research: can Reconfigurable Intelligent Surfaces (RISs) be used to complement satellite-related communications? In the future, we will continue to build more robust and high-quality neural networks for adaptive beamforming algorithms [27,28].

## Data Availability

Data are contained within the article.
